# Chaperone Therapy for Neuronopathic Lysosomal Diseases: Competitive Inhibitors as Chemical Chaperones for Enhancement of Mutant Enzyme Activities

**DOI:** 10.4137/pmc.s2332

**Published:** 2009-05-26

**Authors:** Yoshiyuki Suzuki, Seiichiro Ogawa, Yasubumi Sakakibara

**Affiliations:** 1 International University of Health and Welfare Graduate School, Kita Kanemaru, Otawara, 324-8501 Japan; 2 Department of Biosciences and Informatics, Faculty of Science and Technology, Keio University, Hiyoshi, Kohoku-ku, Yokohama, 223-8522 Japan

**Keywords:** Chaperone, Valienamine, Lysosomal disease, Lysosomal enzyme, β-Galactosidase, β-Glucosidase, G_M1_-gangliosidosis, Gaucher disease

## Abstract

Chaperone therapy is a newly developed molecular approach to lysosomal diseases, a group of human genetic diseases causing severe brain damage. We found two valienamine derivatives, *N*-octyl-4-epi-β-valienamine (NOEV) and *N*-octyl-β-valienamine (NOV), as promising therapeutic agents for human β-galactosidase deficiency disorders (mainly G_M1_-gangliosidosis) and β-glucosidase deficiency disorders (Gaucher disease), respectively. We briefly reviewed the historical background of research in carbasugar glycosidase inhibitors. Originally NOEV and NOV had been discovered as competitive inhibitors, and then their paradoxical bioactivities as chaperones were confirmed in cultured fibroblasts from patients with these disorders. Subsequently G_M1_-gangliosidosis model mice were developed and useful for experimental studies. Orally administered NOEV entered the brain through the blood-brain barrier, enhanced β-galactosidase activity, reduced substrate storage, and improved neurological deterioration clinically. Furthermore, we executed computational analysis for prediction of molecular interactions between β-galactosidase and NOEV. Some preliminary results of computational analysis of molecular interaction mechanism are presented in this article. NOV also showed the chaperone effect toward several β-glucosidase gene mutations in Gaucher disease. We hope chaperone therapy will become available for some patients with G_M1_-gangliosidosis, Gaucher disease, and potentially other lysosomal storage diseases with central nervous system involvement.

## Introduction

Lysosome is one of the cellular organelles where various high molecular endogenous or exogenous compounds are systematically digested under the acidic condition.^1^ This physiological catalytic process is disturbed if mutations occur in one of the genes coding for the hydrolytic enzymes in the lysosome. Celllular dysfunctioin caused by an excessive storage of substrates ensues, and a genetic metabolic disease (lysosomal disease) develops clinically in humans and other animals with neurological and other somatic manifestations. This concept was first proposed for glycogen storage disease type I.^2^

Since the mid-1960s, attempts have been made to the development of therapy for patients with lysosomal diseases. Theorectically enzyme replacement therapy was the most promising approach, and eventually shown to be effective for Gaucher disease patients, the most prevalent metabolic storage disorder of humans.^3^ This approach has been extended to other lysosomal diseases. However, the effect has not been confirmed to brain pathology in patients with neurological manifestations.

G_M1_-gagliosidosis is one of the lysosomal diseases with storage of ganglioside G_M1_, keratan sulfate, and glycoprotein-derived oligosaccharides, presenting clinically with progressive neurological deterioration mainly in infancy and childhood.^4^ This disease has been our major target of research for more than 40 years. We analyzed correlation of phenotypic manifestations with storage compounds,^5^ enzyme activities,^6^,^7^ and enzyme molecules.^8^,^9^ Finally we moved to molecular pathology of β-galactosidase.^10^

In parallel with with these experiments, in the early 1990s, we started molecular analyses of two genetically distinct human disease groups, β-galactosidosis (β-galactosidase deficiency disorders) caused by β-galactosidase gene mutations^4^ and Fabry disease caused by α-galactosidase A gene mutations.^11^ A paradoxical phenomenon was found that galactose itself and anologous low molecular weight competitive inhibitors could serve as chemical chaperones to induce expression of catalytic activities of mutant enzymes after stabilization and successful intracellular transport to the lysosome in the cells. We reported this enhancement first in Fabry disease,^12^,^13^ and then in G_M1_-gangliosidosis^14^ and Gaucher disease.^15^

After our early studies on galactose and 1-deoxygalactonojirimycin (DGJ), we developed new valienamine derivatives, *N*-octyl-4-epi-β-valienamine (NOEV) and *N*-octyl-β-valienamine (NOV) as chemical chaperones for mutant β-galactosidase and β-glucosidase proteins, respectively, to restore the enzyme activity in somatic cells from patients with G_M1_-gangliosidosis and Gaucher disease.^14^–^16^ We hope that this phenomenon will be applied to development of novel molecular therapeutic approach to lysosomal diseases, particularly with severe brain damage, in the near future. In this article we summarize our experimental results of chaperone effect and chaperone therapy mainly on NOEV for G_M1_-gangliosidosis.

## Competitive Inhibitors of Lysosomal Enzymes

### Carbasugar glycosidase inhibitors and related bioactive compounds

**Figure 1.**
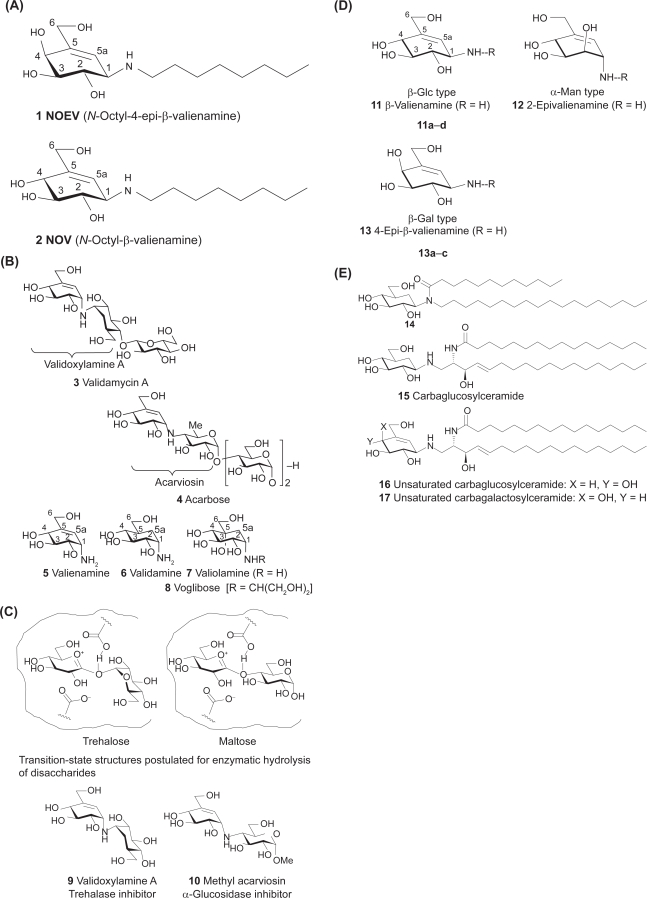
**Structures of valienamines and related compounds. 1A)** *N*-Octyl-4-epi-β-valienamine (NOEV) and *N*-octyl-β-valienamine (NOV). **1B)** Antibiotic validamycin A and α-amylase inhibitor acarbose. Carbaglycosylamine-type α-glucosidase inhibitors: valienamine, validamine, and valiolamine. **1C)** Validoxylamine A and methyl acarviosin are mimicking the postulated transition-state structures for hydrolysis of trehalose and maltose, respectively. **1D)** Some biologically interesting valienamine analogues and N-alkyl derivatives [R = (CH_2_)_n_CH_3_]. **1E)** Biologically active carbaglucosylamide and carbaglucosylceramide, and chemically modified unsaturated derivatives.

Carbasugars, previously known as pseudosugars, are a family of sugar mimics currently attracting interest among researchers in glycobiology and chemistry fields.^17^–^19^ The first example of carba-α-talopyranose was synthesized and called “pseudosugars.”^20^ Later, naturally occurring bioactive carbasugar, 5a-carba-α-D-galactopyranose, was discovered.^21^ Carbasugars are (hydroxymethyl)-branched-chain cyclitols. They are topologically similar to normal sugars particularly in the arrangement of the hydroxyl and hydroxymethyl groups, but have the oxygen atom of the pyranose or furanose ring replaced by methylene. Humans cannot differentiate carbaglucose from true glucose by their taste.^22^ Furthermore carbahexopyranoses exist in structurally stable α- and β-anomer forms which are not interconvertible. Therefore, chemical modification at C-1 positions may be possible, providing biologically interesting compounds. Potent glycosidase inhibitors NOEV **1** and NOV **2** (Fig. 1A) are synthetic 5,5a-unsaturated 5a-carba-β-galacto and glucopyranosylamine derivatives, respectively.

In 1970, agrochemical antibiotic validamycin A **3** and homologues were discovered^23^ and have so far been utilized to control sheath bright disease of rice plant (Fig. 1B). During their structural elucidation, three components were isolated: valienamine **5**, validamine **6,**^24^ and valiolamine **7.**^25^ These compounds are strong α-glucosidase inhibitors themselves. In 1976, α-amylase inhibitor acarbose **4** composed of **5** was discovered^26^ and has been clinically used to control diabetes.

### Structural modification of valienamine

The enzyme inhibitory potency of **4** has been attributed to the core structure of methyl acarviosin **10,** a strong α-glucosidase inhibitor, and considered to be an analogue of transition-state structure postulated for enzymatic hydrolysis of maltose^27^ (Fig. 1C). The potency of **3** has been attributed to strong trehalase inhibition *in vitro* by validoxyl-amine A **9** that mimicks the transition-state structure for hydrolysis of trehalose with trehalase. The valienamine moiety of **9** and **10** holds the pyranoid oxonium ion structures and binds more firmly to the corresponding active sites of enzymes via an imminium ion. Thus, they are competitive inhibitors, explaining a structural correlation between inhibitors and substrates. This knowledge opened up the possibility for development of therapeutically useful carbasugar derivatives. Thus, chemical modification of three components **5–7** was stimulated by successful medical application of **4** (Fig. 1B), leading to a finding of semisynthetic voglibose **8**, *N*-(1,3-dihydroxyprop-2-yl)valiolamine that is fully compatible to **4.**^28^,^29^

One of the authors (SO) became interested in chemical modification of **5** and **6**, and designed some stereoisomers by analogy of the structural relationship between enzyme inhibitors and substrates. However, both the C-1 epimers of **5** and **6** unexpectedly lacked activity against β-glucosidase. Then valienamine-type glycosidase inhibitors **11–13** with β-*gluco*, α-*manno*, and β-*galacto* configurations, expected to be specific inhibitors of the corresponding hydrolases, were synthesized^30^,^31^ (Fig. 1D). However, notable inhibitory activity could not be observed for any of them. Bioassay of the activity was carried out routinely using commercially available glycosidases. Although simple chemical modification of these amines, such as *N*-alkylation, might have improved their potency, the activity of intact amines should be a reliable hallmark for further development of its related compounds. Undoubtedly, biochemical features of **5** and **6** have been important models for further successful development.

### Glycocerebrosidase inhibitors: carbaglycosylceramides

Some glycosylamides are significant immunomodulators.^32^ We prepared some carbasugar analogues, such as compound **14**, as bioactive glycosylamides^33^ (Fig. 1E). This result suggested that the carbohydrate moiety of glycolipids could possibly be replaced to give rise to biologically active carbohydrate mimics.

Then a carbasugar mimic **15** of glucosylceramide was found to be a moderate inhibitor of glucocerebrosidase.^34^ Further attempts were made to prepare valienamine analogues **16** and **17**, carbaglucosyl and carbagalactosylceramides,^35^ which exhibited potent and specific inhibitory activity against the corresponding gluco- (IC_50_, 0.3 μM; bovine liver) and galacto-cerebrosidases (IC_50_, 2.7 μM; bovine liver).

### *N*-Alkyl valienamines, potent β-galactosidase inhibitors

Complex ceramide chains were synthetically inaccessible. We therefore began to modify the structure by introducing a simple substitution of ceramide chain. Replacement with a simple aliphatic chain resulted in an increase of inhibitory activity^36^ (Fig. 1D). Table 1 shows enzyme inhibitory activity of *N*-alkyl derivatives **1, 2, 11a**–**d**, and **13a**–**c** [R=(CH_2_)_n_CH_3_]toward some glycosidases.^19^,^37^ *N*-Octyl derivatives **1** and **2** were most promising for medical application.^38^ Biochemical role of *N*-octyl portion was predicted also by computer-assisted simulation studies (Sakakibara, unpublished data).

### Preparative studies on valienamine type inhibitors

For further development of carbasugar chemistry, simple synthetic precursors are required. Diels-Alder *endo*-adduct **18**(+), (−) of furan and acrylic acid has been useful for this purpose. Preparative intermediates reported in previous studies^19^ could also be provided as optically pure forms from common hexopyranoses^39^ providing a convenient link of carba to true sugars. Optical resolution of racemic **18** could be readily conducted through fractional crystallization of the diastereomeric salt with optically active α-phenylethylamines,^40^ or enantioselective hydrolysis of the racemic 2-chloroethyl epoxy esters by means of pig liver esterase (Sugai et al. unpublished data).

**Figure 2.**
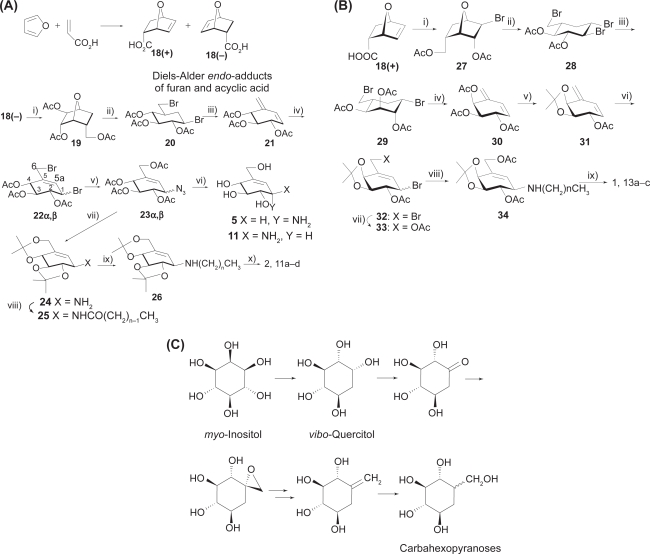
**Synthetic pathways of valienamines and related compounds. 2A)** Synthesis of valienamines and *N*-alkyl derivatives. (i) H_2_O_2_, HCOOH; LAH/THF; Ac_2_O/Pyrd; (ii) HBr/AcOH; (iii) DBU/toluene; (iv) Br_2_/CCl_4_; (v) AcONa/MeO(CH_2_)_2_OH; NaN_3_/DMF; (vi) MeONa; Ph_3_P/MeOH; (vii) MeONa; (MeO)_2_ CMe_2_, TsOH/DMF; Ph_3_P/MeOH; (viii) CH_3_ (CH_2_)_n–1_COCl/Pyrd; (ix) LAH/THF; (x) aq. AcOH; acidic resin, aq. NH_3_. **2B)** Synthesis of *N*-alkyl-4-epi-β-valienamines. (i) Br_2_, Na_2_CO_3_/H_2_O; LAH/THF; Ac_2_O/Pyrd; (ii) HBr/AcOH; (iii) MeONa; aq. H_2_SO_4_; Ac_2_O/Pyrd; (iv) DBU/toluene; (v) MeONa/MeOH; (MeO)_2_ CMe_2_, TsOH/DMF; Ac_2_O/Pyrd; (vi) Br_2_/CCl_4_; (vii) AcONa/MeO(CH_2_)_2_OH; (viii) CH_3_(CH_2_)_n_NH_2_/DMF; (ix) MeONa/MeOH; aq. AcOH; acidic resin, aq. NH_3_. **2C)** Facile transformation of vibo-quercitol into carbahexopyranoses.

Typical routes to valienamines related to NOEV **1** and NOV **2** were briefly described^41^ (Fig. 2A). Optically resolved *endo*-adduct **18**(−) with l-stereochemistry was converted into 1,6-dibromo-1,6-dideoxy-β-carbaglucose **20** through the triol **19**. Elimination of **20** with 1,8-diazabicyclo[5.4.0]undec-7-ene (DBU) gave the conjugate alkadiene **21**, which was transformed into 1,4-dibromides **22α,β**. The primary bromo group was first replaced with an acetate ion and then the secondary one with azide anion to give α,β-mixture of the azide precursors **23α**,**β**. The azido function was reduced with H_2_S or Ph_3_P to give the free bases **5** and **11**.

Effective *N*-alkylation of **11** is important for provision of active compounds. Compound **23β** was protected and reduced (→**24**). The free amine **24** was treated with a series of acid chlorides to give the amides (**25**). Reduction of **25** with lithium aluminum hydride (LAH) (→**26**), followed by deprotection, afforded the *N*-alkyl valienamines (→NOV **2, 11a–d**) in good yields.^36^

NOEV **1** is the 4-epimer of **2**. Preparation of **1** was first conducted cumbersomely by Walden inversion at C-4 of **2** through oxidation of 4-OH, followed by selective reduction.^38^ Alternatively, **18**(+) was converted into the bromo acetate (**27**), which was cleaved with HBr to give the tribromide (**28**) (Fig. 2B). Compound **28** was treated with methoxide and the resulting anhydride was opened and then acetylated to give the dibromide (**29**). Dehydrobromination (→**30**) followed by protection gave the alkadiene **31**. Treatment with bromine gave the 1,4-dibromide (**32**), which was similarly converted into the bromide **33**. Thus **33** was similarly transformed into the β-valienamine **13** with β-*galacto* configuration. Modification of the β-galactose-type valienamine will be achieved by direct displacement of bromide **33** with alkyl amine (→**34**). The substitution reaction selectively occurs as expected by neighboring assistance of the 2-acetoxyl or through direct S_N_2 fashion to afford, after deprotection, *N*-alkyl-4-epi-β-valienamines (**13a–c**) including NOEV.^37^

Transition-state type glycosidase inhibitor valienamines have thus been recognized as desirable carbohydrate mimics for designing new glycosidase inhibitors. Current technical difficulty is comparative inaccessibility to the carbahexose skeletons. Recently, we identified some routes to carbasugars through chemical transformation of (−)-*vibo*-quercitol derived from *myo*-inositol by biogenesis (Fig. 2C).^42^,^43^ This route establishes a link between naturally abundant cyclitols and chiral carbasugars.

### Future aspect

Carbaglycosylamines are chemically stable (hydroxymethyl) aminocylitols and expected to play roles as non-hydrolyzable mimics of glycopyranosylamines.^43^,^44^ Diverse modification of the biochemical and topologic nature of carbaglycosylamines may be achieved by substitution at the anomeric position, unsaturation at C-5 and C-5a, and hydroxylation at C-5 and/or C-5a, leading to improvement of biological function. As shown by the inhibitory activity of the *N*-alkyl derivatives **11a–d** and **13a**–**c** of β-valienamines, the inclusion of hydrophobic *N*-alkyl chains is likely to contribute to improvement of its potential significance. Furthermore, additional modification of their physicochemical nature is advisable for the purpose of generating strong binding to active sites of enzyme or peptide molecules.

## β-Galactosidosis: Genetic Human Disorders Caused by β-Galactosidase Gene Mutations

Lysosomal β-galactosidase (EC 3.2.1.23), encoded by a gene *GLB1* (3p21.33), catalyzes hydrolysis of ganglioside G_M1_ and related glycoconjugates such as oligosaccharides derived from glycoproteins and keratin sulfate in human somatic cells. Allelic mutations of the gene result in excessive storage of the substrates in various cells and tissues.

G_M1_-gangliosidosis (OMIM 230500) is expressed clinically as generalized neurosomatic disease in children (infantile form, juvenile form), and rarely in adults (adult form), caused by widespread abnormal storage of ganglioside G_M1_, mucopolysaccharide keratin sulfate and glycoprotein-derived oligosaccharides in the central nervous system, skeletal system, and other tissues and visceral organs. Specific gene mutations are known for each clinical form.^45^ Morquio B disease (OMIM 253010) is another clinical phenotype presenting with generalized skeletal dysplasia without neurological involvement. Again specific gene mutations different from those in G_M1_-gangliosidosis have been identified.^46^ More than 100 gene mutations are collected, and successful gene diagnosis is well established using restriction enzymes specific to individual mutations.^4^

At present only symptomatic therapy is available for the brain lesion in human G_M1_-gangliosidosis patients. Enzyme replacement therapy is currently in use for clinical practice for Gaucher disease, Fabry disease and other lysosomal diseases. However, the beneficial effect has not been confirmed for the brain damage, although general somatic signs and symptoms are clearly improved by continuous enzyme replacement therapy.^47^ Secretion of feline β-galactosidase was reported in the transfected cell culture system, but the effect on the central nervous system was not shown.^48^

After several years of basic investigations mainly for mutant α-galactosidase A in Fabry disease, we proposed chemical chaperone therapy for brain pathology in G_M1_-gangliosidosis, using an *in vitro* enzyme inhibitor *N*-octyl-4-epi-β-valienamine (NOEV) (**1**; Fig. 1A), a chemical compound newly produced by organic synthesis described above,^38^ as a potent stabilizer of mutant β-galactosidase in somatic cells from patients with this disorder.^14^

## Genetic Metabolic Diseases: Molecular Pathology and an Approach to Possible Molecular Therapy

Molecular pathology of inherited metabolic diseases can be classified into the following three major conditions.^49^

- Biosynthetic defect of the protein in question. Mutant enzyme is not synthesized, and accordingly rescue of the protein is not possible.
- Defect of biological activity. In spite of normal biosynthesis, the protein does not maintain biological activity because of its structural abnormality. There is no possibility to restore the biological activity of this molecule.
- Unstable mutant protein with normal or near-normal biological activity under appropriate environmental conditions. The mutant protein has normal biological function in its mature form. However, it is unstable and rapidly degraded immediately after biosynthesis. The protein function is expected to be restored if the molecule is somehow stabilized and transported to the cellular compartment where it is expected to exhibit biological activity; the lysosome in the case of lysosomal enzyme.

We tested these possibilities first in Fabry disease, and found some mutant enzyme proteins were unstable at neutral pH in the endoplasmic reticulum/Golgi apparatus, and rapidly degraded because of inappropriate molecular folding.^50^ Addition of galactose in the culture medium of lymphoblasts from Fabry patients and COS-1 cells expressing mutant enzyme proteins surprisingly induced a high expression of α-galactosidase A activity.^12^ However, a high concentration of galactose was necessary for treatment of these enzyme-deficient cells in culture. We concluded that a long-term treatment with galactose at this high dose was not realistic, although a short-term human experiment was reported on the beneficial cardiac function in a case of Fabry disease.^51^ Accordingly we searched for other compounds that could enhance the enzyme activity in mutant cells. As stated above, DGJ was found to be effective for stabilization and high expression of the enzyme activity.^13^,^52^ DGJ showed the chaperone effect mainly on mutant α-galactosidae A. Its activity toward mutant β-galactosidase was 50-fold lower in a culture system experiment.^53^

After subsequent extensive molecular analysis we reached the following conclusion.^49^ A substrate analogue inhibitor binds to the misfolded mutant lysosomal protein as a kind of molecular chaperone (chemical chaperone), to achieve normal molecular folding at the endoplasmic reticulum/Golgi compartment in somatic cells, resulting in formation of a stable molecular complex at neutral pH. The protein-chaperone complex is safely transported to the lysosome, where it dissociates under the acidic conditions, the mutant enzyme remains stabilized in its normal folding, and its catalytic function is expressed (see below; Fig. 4).

## Therapeutic Approach to Brain Pathology by Chemical Chaperones

### New chaperones: valienamine derivatives

We had particular interest in primary neuronopathic lysosomal diseases. Accordingly after studies on galactose and DGJ for α-galactosidase A, we started an extensive search for specific compounds for β-galactosidase. No commercially available compound was found as bioactive chaperone. Fortunately we came across two synthetic valienamine derivatives: β-galactosidase inhibitor *N*-octyl-4-epi-β-valienamine (NOEV) and β-glucosidase inhibitor *N*-octyl-β-valienamine (NOV) (**1** and **2**; Fig. 1A). NOV was the first compound synthesized as a glucocerebrosidase inhibitor,^36^ and subsequently NOEV was synthesized by epimerization of NOV.^37^ They are specific competitive inhibitors of β-galactosidase and β-glucosidase, respectively, In our laboratory NOEV studies moved faster than NOV simply because of accumulation of more experimental data and clinical materials for β-galactosidase and G_M1_-gangliosidosis.

NOEV is a potent inhibitor of lysosomal β-galactosidase *in vitro*. It is stable and soluble in methanol or DMSO. The hydrochloride salt is freely soluble in water. Molecular weight is 287.40. IC_50_ is 0.125 μM toward human β-galactosidase.^14^

**Figure 3.**
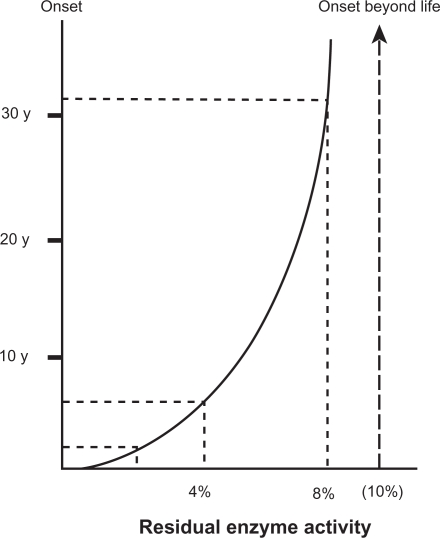
**Correlation between residual β-galactosidase activity and clinical onset.** The amount of residual enzyme activity shows positive parabolic correlation with the age of onset in various phenotypic forms of β-galactosidase deficiency disorders. The enzyme activity is generally less than 3% of the control mean in infantile G_M1_-gangliosidosis, 3%–6% in juvenile G_M1_-gangliosidosis, and more than 6% in late onset (adult/chronic) G_M1_-gangliosidosis and Morquio B disease. At least 10% of normal enzyme activity is necessary for washout of the storage substrate. The age of onset in patients expressing enzyme activity above this level is theoretically beyond the human life span. This figure is based on the enzyme assay results using cultured skin fibroblasts and a synthetic fluorogenic substrate 4-methylumbelliferyl β-galactopyranoside. In this calculation, for technical reasons, substrate specificity is not taken into account, although mutant enzymes show different spectrum in G_M1_-gangliosidosis and Morquio B disease.

NOEV is 50-fold more active than DGJ in chaperone effect on mutant human β-galactosidase in G_M1_-gangliosidosis. Our calculations suggest that at least 10% of normal enzyme activity is necessary for washout of the storage substrate in lysosomal diseases (Fig. 3). The age of onset in patients expressing enzyme activity above this level is theoretically beyond the human life span. The same calculation was reported on some other lysosomal diseases.^54^ We anticipate that the effective NOEV concentrations in human cells and animal tissues are much lower than the IC_50_ calculated *in vitro*, based on the results of tissue concentration after oral NOEV administration in experimental model mice.^55^ In fact, NOEV is effective at the IC_50_ concentration in the culture medium for enhancement of mutant enzyme activity.^16^ We hope it will be clinically used as a specific enzyme enhancer without exerting inhibitory effect in the cells.

### Chaperone experiments on culture cells

About one-third of the cultured fibroblast strains from G_M1_-gangliosidosis patients responded to NOEV; mainly from juvenile form and some infantile form patients. The effect is mutation-specific.^16^ The R457Q mutant responded to NOEV maximally at 0.2 μM, and the R201C or R201H mutant at 2 μM. The mouse fibroblasts expressing mutant human β-galactosidase showed essentially the same results.^53^ Molecular interaction between the chaperone and mutant protein depends on the structural modification of the mutant enzyme protein (Sakakibara et al. unpublished data). Addition of ganglioside mixture in the culture medium increased intracellular G_M1_ in the R201C cells causing juvenile G_M1_-gangliosidosis.^14^ This storage was almost completely prevented by NOEV.

### Chaperone experiments on genetically engineered G_M1_-gangliosidosis model mice

For animal studies we developed a knockout (KO) mouse strain with complete deficiency of β-galactosidase,^56^ and then a transgenic (Tg) strain based on KO, expressing human R201C mutation (4% of the enzyme activity in the brain from wild-type mice).^14^ Both showed neurological deterioration with some difference in severity. Life span was 7–10 months for KO and 12–18 months for Tg. Neuropathology corresponded to the clinical severity.^14^ Short-term oral NOEV administration resulted in significant enhancement of the enzyme activity in all the R201C mouse tissues examined, including the brain.^14^ Immunohistochemstry revealed an increase in β-galactosidase activity and decrease in G_M1_ and G_A1_ storage.

Oral NOEV treatment for the R201C Tg mouse showed an increase of the NOEV content in the brain in parallel with β-galactosidase activity, and G_M1_ storage decreased.^55^ NOEV disappeared rapidly, within a few days after withdrawal. In this study we tried a new scoring system for neurological assessment^57^ (Table 2). Treatment at the very early clinical stage (2 months) resulted in a positive clinical effect within a few months, although complete arrest or prevention of disease progression was not achieved under this experimental condition (Table 3). The latency before the clinical effect was longer if the therapy was started in the late symptomatic stage (6 months). We concluded that NOEV treatment at the early stage of disease is mandatory for prevention of the brain damage.

This result indicated the following sequence of events in the mouse brain.^49^ After oral administration, NOEV goes directly into the bloodstream without intestinal digestion, is delivered to the brain through the blood-brain barrier, and restore the mutant β-galactosidase activity, resulting in substrate digestion and clinical improvement. No specific adverse effects have been observed for at least 6 months of continuous oral administration. For achievement of clinical drug development, however, we need to study further possible adverse effects and to establish the optinal dose and frequency of administration in order to achieve the best clinical effect.

## Molecular Mechanisms of Chaperone Effect in Lysosomal Disease

**Figure 4.**
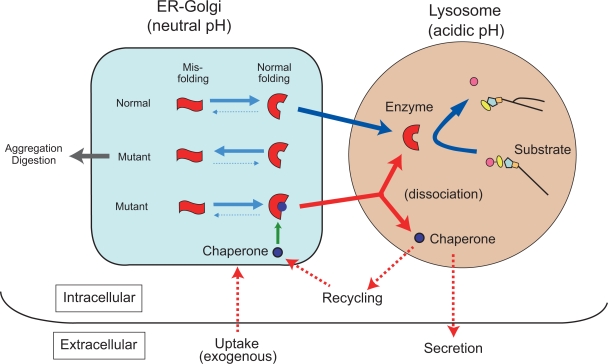
**Postulated molecular events between mutant enzyme molecules and chaperone compounds.** Mutant enzyme protein is unstable in the endoplasmic reticulum (ER)-Golgi compartment at neutral pH, and rapidly degraded or aggregated possibly to cause ER stress. An appropriate substrate analogue inhibitor binds to misfolded mutant protein as chemical chaperone at the ER-Golgi compartment in somatic cells, resulting in normal folding and formation of a stable complex at neutral pH. The protein-chaperone complex is safely transported to the lysosome. The complex is dissociated under the acidic condition and in the presence of excessive storage of the substrate. The mutant enzyme remains stabilized, and express catalytic function. The released chaperone is either secreted from the cell or recycled to interact with another mutant protein molecule. These molecular events have been partially clarified by analytical and morphological analyses, and computer-assisted prediction of molecular interactions.

As described above, β-galactosidase gene mutations result in excessive accumulation of substrates and various clinical phenotypes: G_M1_-gangliosidosis and Morquio B disease. Single base substitutions do not necessarily lead to a complete loss of enzyme function. However, the enzyme activity is not always expressed even if the potential catalytic function is not completely lost, simply because of intracellular instability of the mutant enzyme molecule due to inappropriate or incorrect protein folding. Molecular pathology of this type occurs at least in one-third of the patients with β-galactosidase deficiency.^16^ Chemical chaperone corrects the molecular abnormality of this type, and assists intracellular transport to the lysosome, finally releasing the mutant enzyme as a stable bioactive protein (Fig. 4).

We postulated that enzyme-chaperone binding would become less strong under the acidic condition in the lysosome. Then the mutant enzyme molecule is released and stable catalytic activity appears. However, the precise mechanism of this NOEV effect is unknown at present. We therefore started computational analysis for prediction of molecular interactions between the β-galactosidase protein and the chaperone compound NOEV.

**Figure 5.**
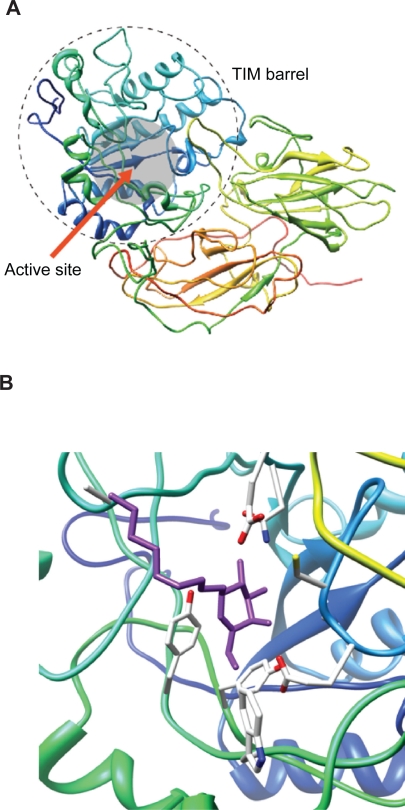
**Computationally predicted structure of β-galactosidase and its conformation of β-galactosidase and NOEV complex. 5A**) Sequence identity in the front part was enough to reconstruct its structure and formed a typical TIM barrel domain that is generally found in glycoside hydrolases. In alignment of this part, active residues of both human and Penicillium sp. β-galactosidase molecules were well matched. **5B**) Docking of β-galactosidase and NOEV was performed. In the complex of β-galactosidase and NOEV in pH7, the ring part of NOEV was settled in the active pocket. Oxygen of a glutamic acid in β-galactosidase and hydroxyl of amido in NOEV interacted via hydrogen bonding.

First, the three-dimensional structure of human enzyme was predicted employing a homology modeling method 3D-JURY,^58^,^59^ because the structure of this enzyme is not yet available. *Penicillium sp.* β-galactosidase was used as the template structure for homology modeling, and the predicted structure of human β-galactosidase has been obtained as shown in Figure 5A.

Second, plausible conformation of β-galactosidase-NOEV complex was determined in support of AUTODOCK4.^60^ The conformation was subjected to further structural optimization. The result of the complex structure was successfully computed by AUTODOCK4 (Fig. 5B).

Third, the binding free energy of the two molecules in the complex was calculated by using AMBER9.^61^ The computed binding free energy was −20.08 (kcal/mol) at pH 7.

Fourth, we calculated the effect of low pH in the lysosome on the binding affinity between the β-galactosidase and NOEV molecules. The low pH effect was represented as protonation of charged residues estimated by PROPKA.^62^ The computed binding free energy at pH 5 was −18.06 (kcal/mol); higher than that at pH 7. This result indicates that affinity between β-galactosidase and NOEV is weakened at pH 5 compared with that at pH 7. Consequently, we concluded that (1) the enzyme-NOEV complex has lower free energy than the unbound enzyme, and (2) protonation of an active site residue causes free energy change consistent with the chemical chaperone hypothesis.

## Conclusion

This new therapeutic strategy (chaperone therapy) is in principle applicable to all lysosomal diseases, if a specific compound is developed for each enzyme in question. We have already confirmed the effect in Fabry disease, G_M1_-gangliosidosis, and Gaucher disease. Other related diseases also are currently studied by other investigators.^63^,^64^ Theoretically this principle can be applied to all other lysosomal diseases. Furthermore, there may well be other genetic diseases to be considered, if molecular pathology in somatic cells has been clarified in detail. We hope studies in this direction will disclose a new aspect of molecular therapy for inherited metabolic diseases with central nervous system involvement in the near future.

## Figures and Tables

**Table 1. A:** Inhibitory activity [*K_i_* (μM)] of some *N*-alkyl-4-epi-β-valienamines against three glycosidases.

**Compound**		**n**	**β-Galactosidase^a^**	**α-Galactosidase^b^**	**β-Glucosidase^c^**
β-Galacto type	**1**	7	0.87	3.1	3.1
	**13a**	5	2.3	2.7	1.2
	**13b**	9	0.13	1.9	2.5
	**13c**	11	0.01	4.4	0.87

^a^ Bovine liver,

^b^ Green coffee beans,

^c^ Almonds.

**Table 1. B:** Inhibitory activity [*K*_i_ (μM)] of some *N*-alkyl-β-valienamines against glucocerebrosidase.

**Compound**		**n**	**Glucocerebrosidase^d^**
β-Gluco type	**2**	7	0.03
	**11a**	5	0.3
	**11b**	9	0.07
	**11c**	11	0.12
	**11d**	13	0.3

^d^ Mouse liver.

**Table 2.** Neurological examination of genetically engineered G_M1_-gangliosidosis model mice. Each test is performed with semi-quantitative time, space, and movement parameters. See Ichinomiya et al.^57^ for details.

**Gait:** (hip, knee, spine, and shivering) Score 0: Normal. Score 1: Slight gait disturbance. Score 2: Marked gait disturbance. Score 3: Marked staggering and shaking; gait impossible. **Posture: forelimb** (paralysis, deformity) Score 0: Normal. Score 1: Starting gait difficult and clumsy. Score 2: Dragging limbs; inversion of dorsum pedis. Score 3: Complete paralysis; no spontaneous movement. **Posture: hind limb** (abduction, extention, posture) Score 0: Normal; smooth joint flexion and extension. Score 1: Slight hip abduction, external rotation, and knee extension; wide-based. Score 2: Severe hip abduction, external rotation, and knee extension; wide-based. Score 3: No spontaneous movement. **Trunk** (deformity) Score 0: Normal. Score 1: Slight back hump. Score 2: Moderate back hump. Score 3: Severe back hump. **Tail** (posture, stiffness) Score 0: Normal Score 1: Slight stiffness and elevation. Score 2: Severe stiffness and elevation. Score 3: Severe stiffness and elevation with persistent deformity. **Avoiding response** (pinching tail root with forceps for one second) Score 0: Strong rejection, avoidance, and squeaking. Score 1: Slight decrease of response. Score 2: Trunk torsion; hind limb extension. Score 3: No response. **Rolling over** (turning the tail root three times to left and right) Score 0: Extending four limbs, resisting passive rolling. Score 1: Slow passive rolling; prompt recovery. Score 2: Markedly slow passive rolling; delayed recovery. Score 3: Posture change impossible; slow body movement. **Body righting acting on head** (response to vertical hanging, head down by holding tail tip, and quick upward movements) Score 0: Strong upward righting reaction of the head. Score 1: Slight decrease in response. Score 2: Marked decrease in response. Score 3: No response; trunk rotation only. **Parachute reflex** (response to vertical hanging, head down by holding tail tip, and quick downward movement, three times, within 30 sec) Score 0: Extension and abduction of hind limbs; continuous knee extension. Score 1: Slight decrease in response; intermittent knee extension. Score 2: Marked decrease in response; flexion and adduction of hind limbs; slow movements. Score 3: No response; continuous flexion and adduction of hind limbs. **Horizontal wire netting** (stepping through interstice during walking on horizontal wire netting) Score 0: No stepping into interstice. Score 1: 21–30 sec before stepping into interstice. Score 2: 11–20 sec before stepping into interstice. Score 3: 0–10 sec before stepping into interstice. **Vertical wire netting** (clinging and holding body on vertical wire netting) Score 0: Stay for 30 sec. Score 1: Stay for 21–30 sec before falling. Score 2: Stay for 11–20 sec before falling. Score 3: Stay for 0–10 sec before falling.

**Table 3.** Effect of NOEV on G_M1_-gangliosidosis Tg mice. Experimental mice were orally fed with water (0 mM NOEV) or NOEV solution (1 mM) for 6 months. Total assessment scores were calculated for each group. Value = mean ± SEM (n); ns = statistically not significant. For details see Suzuki et al.^55^

**NOEV**	**0 mM**	**1 mM**	**t test**
2 months	1.72 ± 0.19 (32)	1.53 ± 0.17 (17)	ns
3 months	2.18 ± 0.38 (11)	1.77 ± 0.24 (17)	ns
4 months	2.53 ± 0.29 (19)	2.06 ± 0.23 (16)	ns
5 months	3.35 ± 0.33 (17)	2.40 ± 0.32 (15)	p < 0.05
6 months	3.90 ± 0.31 (30)	2.81 ± 0.25 (16)	p < 0.05
7 months	4.88 ± 0.57 (17)	3.43 ± 0.20 (14)	p < 0.05

## Abbreviations

NOEV: *N*-octyl-4-epi-β-valienamine;
NOV: *N*-octyl-β-valienamine;
DGN: 1-deoxygalactonojirimycin;
DBU: 1,8-diazabicyclo[5.4.0]undec-7-ene;
LAH: lithium aluminum hydride;
KO: knockout;
Tg: transgenic.
